# Literacy and blood pressure – do healthcare systems influence this relationship? A cross-sectional study

**DOI:** 10.1186/1472-6963-8-219

**Published:** 2008-10-23

**Authors:** Benjamin J Powers, Maren K Olsen, Eugene Z Oddone, Carolyn T Thorpe, Hayden B Bosworth

**Affiliations:** 1 Center for Health Services Research in Primary Care, Durham VAMC, Durham NC, USA; 2 Department of Medicine, Division of General Internal Medicine, Duke University, Durham NC, USA; 3 Department of Biostatistics and Bioinformatics, Duke University, Durham NC, USA; 4 Department of Psychiatry and Behavioral Sciences & Center for Aging and Human Development, Duke University, Durham NC, USA

## Abstract

**Background:**

Limited literacy is common among patients with chronic conditions and is associated with poor health outcomes. We sought to determine the association between literacy and blood pressure in primary care patients with hypertension and to determine if this relationship was consistent across distinct systems of healthcare delivery.

**Methods:**

We conducted a cross-sectional study of 1224 patients with hypertension utilizing baseline data from two separate, but similar randomized controlled trials. Patients were enrolled from primary care clinics in the Veterans Affairs healthcare system (VAHS) and a university healthcare system (UHS) in Durham, North Carolina. We compared the association between literacy and the primary outcome systolic blood pressure (SBP) and secondary outcomes of diastolic blood pressure (DBP) and blood pressure (BP) control across the two different healthcare systems.

**Results:**

Patients who read below a 9^th ^grade level comprised 38.4% of patients in the VAHS and 27.5% of the patients in the UHS. There was a significant interaction between literacy and healthcare system for SBP. In adjusted analyses, SBP for patients with limited literacy was 1.2 mmHg lower than patients with adequate literacy in the VAHS (95% CI, -4.8 to 2.3), but 6.1 mmHg higher than patients with adequate literacy in the UHS (95% CI, 2.1 to 10.1); (p = 0.003 for test of interaction). This literacy by healthcare system interaction was not statistically significant for DBP or BP control.

**Conclusion:**

The relationship between patient literacy and systolic blood pressure varied significantly across different models of healthcare delivery. The attributes of the healthcare delivery system may influence the relationship between literacy and health outcomes.

## Background

Over 90 million adult Americans lack the literacy skills to effectively function in the current healthcare environment [1] – a number that has not changed significantly in the past 10 years [2]. Low health literacy is found in many different healthcare settings [3,4] and is most common in older patients, those with lower education levels, immigrants, and racial minorities [5]. Prior research has supported the association between literacy and disease knowledge, utilization of preventative services, hospitalization, overall health status, chronic disease control, and mortality in elderly adults [6-8]. Due to a growing body of evidence regarding these associations, literacy has been deemed a national priority [1,9,10].

We examined the relationship between literacy and blood pressure (BP) in primary care patients with hypertension. Hypertension affects approximately 65 million individuals in the United States [11,12] and is an important modifiable risk factor for cardiovascular disease, stroke, and renal disease [13,14]. In spite of effective therapies, only 37% of patients with hypertension achieve their target BP, well below the goal of 50% set by Healthy People 2010 [15]. Hypertension control often requires patients to engage in multiple complex tasks including medication adherence, frequent medical visits, and diet and lifestyle modification and these tasks may be more difficult for patients with limited literacy. The extent to which literacy is associated with BP control among hypertensive patients is uncertain. Williams et al. found that patients with hypertension and limited literacy at two public hospitals were significantly less knowledgeable about their hypertension care than patients with adequate literacy and had a 6 mmHg higher SBP; however this blood pressure difference was not statistically significant [16]. Rothman et al. reported that literacy was an important predictor of improved glycemic control for diabetic patients enrolled in a disease management trial; however there were no significant differences between low and higher literacy patients in SBP either at baseline or at the conclusion of the trial. [17]

Although a great deal of research has focused on defining and understanding the relationship between literacy and health, it is less clear how to improve outcomes for patients with limited literacy. If adequate literacy is required for a patient to successfully navigate encounters with a healthcare delivery system, the impact of low literacy may be exacerbated or mitigated by the features of that system. This is supported by the finding that patients with limited literacy particularly benefit from disease management interventions that address the increased challenges of limited literacy and improve patient self-management skills [17,18]. However, it is not known whether the relationship between literacy and health outcomes is consistent across different systems of healthcare delivery.

To evaluate this, we examined a racially and economically diverse sample of patients who received primary care for hypertension in one of two healthcare systems in Durham, North Carolina. Patients received care in either the Veterans Affairs health system (VAHS), an integrated delivery system with affordable prescription medications for all of its enrollees [19], or a university health system (UHS) where available services and medication coverage varied according to insurance status. The goals of this study were to 1) describe the relationship between literacy and BP in patients with hypertension and 2) determine if this relationship is consistent between the two distinct healthcare systems.

## Methods

### Patients selection

We pooled data from patient interviews performed at the time of enrollment for two separate randomized controlled trials to improve BP control. All patients provided written informed consent and both studies were approved by their respective institutional review boards. The patients who comprised our VAHS sample were all enrolled in the Veteran Study to Improve the Control of Hypertension (V-STITCH), which was conducted in three Durham VA Medical Center primary care clinics [20]. This trial tested a self-management support intervention delivered by a nurse over the phone as well as a computer decision support system for primary care providers. All primary care providers were either general internists with a faculty appointment at Duke Division of General Internal Medicine or Physician Assistants under the supervision of the faculty physicians. Potential subjects were identified through the facility's electronic medical records and were required to have a diagnosis of hypertension based on an outpatient ICD-9 code of 401.0, 401.1, or 401.9, and a filled prescription for hypertensive medication in the previous year. Of the initial pool of 4017 potential veteran subjects, the research assistants approached 816 patients. Because recruitment occurred at the primary care visit, these patients were selected from the available pool based on the timing of a scheduled primary care appointment. Patients were recruited consecutively until the target sample size for the primary study hypothesis was achieved. Of the 816 approached, 190 refused, 38 patients were excluded, and a total of 588 patients were enrolled between March, 2002 and April, 2003.

Using similar recruitment strategies and inclusion/exclusion criteria, a second sample of hypertensive individuals was enrolled in the Take Control of Your Blood pressure (TCYB) study [21], and comprised our entire sample of UHS patients. This trial tested a self-management support intervention delivered by a nurse over the phone as well as patient home blood pressure monitoring to improve blood pressure. The patients were recruited from two Duke University Health System primary care clinics: Duke General Internal Medicine (DGIM) and Duke Outpatient Clinic (DOC). Patients followed in the Duke General Internal Medicine clinic were cared for by a mix of faculty general internists in the Duke Division of General Internal Medicine (approximately 83%) and Duke Department of Internal Medicine residents under the supervision of the faculty (approximately 17%). Patients at the Duke Outpatient Clinic were cared for almost entirely by resident providers in the Duke Department of Internal Medicine with all care supervised by faculty physicians. Although the patient characteristics differed between the two clinics, both clinics were part of the same health system with similar access to laboratory, radiology, pharmacy, and specialist referral services, and were therefore combined as representing a common system of healthcare delivery. Of the pool of 7646 potentially eligible patients, the research assistants mailed letters explaining the study to 1692 patients with upcoming primary care appointments. Patients who did not call to opt out of the study were approached at the time of their primary care visit for enrollment and baseline interview. A total of 635 patients refused, 236 were excluded, and 636 were enrolled between May, 2004 and December, 2005.

Reasons for exclusion were similar between the two studies and included: spouse participating in the study; not living in a surrounding eight county catchment area; receiving kidney dialysis; recipient of an organ transplant; planning a pregnancy; hospitalization for stroke, myocardial infarction (MI), coronary artery revascularization in the prior 3 months; diagnosis of metastatic cancer or dementia; residence in a nursing home or receiving home healthcare; difficulty speaking or understanding English; or severe hearing or speech impairment. All measures reported were obtained in the baseline face-to-face interview with the research assistant except for blood pressure and insurance status which are described below. With the exception of the literacy assessment, all questions were read aloud to patients by the research assistant.

### Outcome – blood pressure

Blood pressure readings were abstracted from the individuals' medical record at the time of study entry. For both studies, clinic nurses using standard automated devices systematically obtained the patient's resting seated BP prior to their visit with the primary care provider. Only 8.6% of patients had more than one BP reading available from the day of enrollment; in these cases we used the minimum systolic and diastolic for the baseline BP as we believed this would most likely be the BP reading used by the primary care provider for decision making. Our results for the adjusted analyses were unchanged when using the mean SBP rather than the minimum SBP. There was no formal review of the clinic BP measurements by study personnel for quality control. We chose to use SBP as the primary outcome for the adjusted analysis because it is both a more important risk factor for cardiovascular disease and more difficult to control than diastolic BP [12]. BP control and DBP were evaluated as secondary endpoints. BP control was defined according to Joint National Committee 6 (JNC 6) criteria of BP <140/90 or <130/85 for diabetics [22], as the updated JNC 7 was not published until enrollment was complete for the V-STITCH study.

### Independent variables

#### Literacy assessment

The Rapid Estimate of Adult Literacy in Medicine (REALM) was used to measure literacy [23]. Patients read aloud from a 66-item list of medical terms arranged in increasing difficulty and the measure is scored as a count of correctly pronounced words with a raw sore that can be converted to reading grade estimates. The REALM has high criterion-related validity compared to longer literacy measures [24,25]. Literacy was analyzed as a dichotomous variable with limited literacy defined as REALM score, 0–60 (<9th grade level) and adequate literacy defined as REALM score 61–66 (≥ 9th grade level). This operationalization was based on prior convention and is consistent with findings correlating literacy and mortality using this categorization [8].

#### Additional covariates

The following patient demographic information was collected in both healthcare systems by patient self-report at the time of enrollment: age, gender, race, marital status, education level, presence of diabetes, and financial situation. Race was dichotomized as white or non-white; Hispanic patients were categorized as non-white. Patients were asked to categorize their highest level of education according to the following categories: 0–9th grade, 10–12th grade, some college or vocational school, or college graduate. Financial situation was assessed by asking patients to describe their current finances by one of the following four categories: 1.) enough money after paying bills for special things; 2.) enough to pay the bills, but not purchase extra things; 3.) enough money to pay bills by cutting back on things; or 4.) difficulty paying bills no matter what was done. Patients who reported either of the last two answers were categorized as inadequate income [26].

Self-reported medication adherence was assessed using a four-item measure based on the Morisky scale [27]. Patients were asked to respond as strongly agree; agree; disagree; or strongly disagree to the following four statements: 1.) I sometimes forget to take my blood pressure medicine; 2.) I am sometimes careless about taking my blood pressure medicine; 3.) when I feel better, I sometimes stop taking my blood pressure medicine; 4.) if I feel worse when I take the blood pressure medicine, sometimes I stop taking it. A summary binary variable was created by coding those who responded strongly agree or agree to any of the four questions as 1 (nonadherent); otherwise, patients received a value of 0 (adherent) [28]. Participants were asked if they currently exercise or participate in an active physical sport. Similarly, patients were asked to report their current smoking status. The response choices for these two health behaviors were yes or no. Patients' view of their providers' communication behavior was assessed using the 3-item Participatory Decision Making survey [29], with scores ranging from 3 to 30 and higher scores indicating providers were more likely to involve patients in decision making.

Health insurance information was collected for patients in the UHS from the Duke University Health System billing database. Patients' insurance was categorized as Medicare, Medicaid, commercial, or uninsured. Because non-VA insurance coverage has little effect on out of pocket healthcare expenses for patients in the VA [19], no additional insurance information was collected for VAHS patients.

#### Analyses

Descriptive statistics were calculated for each variable and are presented by healthcare system. To test for differences between healthcare systems we used chi-square for categorical variables and Wilcoxon rank-sum for continuous variables. Multiple linear regression was performed to determine the relationship between literacy and healthcare system with the primary outcome SBP after controlling for potential confounders. We included the interaction term of literacy and health system in the model to test the hypothesis that the association between literacy and SBP may differ across healthcare systems. We adjusted for the following demographic factors that we hypothesized could be related to SBP: age, race, marital status, education level, and adequacy of income. Gender was excluded from the analysis because it was confounded with health system; 98% of the VA population was male. We included diabetic status in the model because this variable may influence the treatment goals for patients with hypertension. Medication adherence, smoking, and exercise were included based on their potential relationship with SBP. Because of the many different physician providers, we included participatory decision making score in the model as an attempt to adjust for physician level differences.

In addition to the primary outcome of SBP, we evaluated the relationship between literacy and DBP using methods identical to those described above for SBP. We also examined the relationship between literacy and healthcare system on the outcome BP control using logistic regression models with and without adjustment for the covariates included in the linear regression models described above. Two sided p-values were used for all analyses with alpha set at 0.05 for rejecting the null hypothesis. All statistical analyses were performed using SAS software, version 9.1 (SAS Institute, Inc., Cary, North Carolina).

## Results

The combined study sample consisted of 1224 patients from the VAHS (n = 588) and UHS (n = 636). Participants' mean age was 62.3 years (SD, 11.9; range, 21 to 92). The sample was evenly balanced between white (52.5%) and non-white (47.2%) patients. The majority (94.5%) of non-white patients identified their race as black. Limited literacy (REALM score indicating below 9^th ^grade reading level) was present in 38.4% of the VAHS sample (n = 226 patients) and 27.6% (n = 175 patients) of the UHS sample.

Patient characteristics are listed according to healthcare system in Table 1. A higher proportion of patients in the VAHS had limited literacy compared to patients in the UHS (38.4% vs. 27.5% respectively). In addition, patients in the VAHS were more likely to be older, male, white, married, have lower educational level, smoke tobacco, and not exercise when compared with patients in the UHS. The mean blood pressure in the VAHS was 138.4/75.5 mmHg (SD, 17.6/11.4) compared to 135.6/77.9 mmHg (SD, 20.5/11.0) in the UHS.

**Table 1:** Characteristics of study participants by healthcare system

	**Healthcare System**				
** Descriptive Characteristic **	** VAHS ** ** (N = 588) **		** UHS ** ** (N = 636) **		** p-value **

					
** REALM Score, N (%) **					
< 9^th ^grade	226	(38.4)	175	(27.5)	<0.001
≥ 9^th ^grade	343	(58.3)	461	(72.5)	
					
**Age, years (SD)**	63.4	(11.4)	61.2	(12.3)	0.002
					
**Gender, N (%)**					
Female	10	(1.7)	420	(66.0)	<0.001
Male	578	(98.3)	216	(34.0)	
					
** Race, N (%) **					
Non-white	250	(42.5)	328	(51.6)	0.002
White	335	(57.0)	308	(48.4)	
					
** Married, N (%) **					
No	189	(32.1)	314	(49.4)	<0.001
Yes	399	(67.9)	320	(50.3)	
					
** Highest Education Level, N (%) **					
0–9th Grade	75	(12.8)	55	(8.6)	<0.001
10th–12th Grade	224	(38.1)	176	(27.7)	
Some College/Vocational	145	(24.7)	160	(25.2)	
College Graduate	144	(24.5)	244	(38.4)	
					
** Diabetes, N (%) **					
No	352	(59.9)	404	(63.5)	0.17
Yes	234	(39.8)	228	(35.8)	
					
** Inadequate Income, N (%) **					
No	456	(77.6)	511	(80.3)	0.25
Yes	127	(21.6)	121	(19.0)	
					
** Self-Reported Medication Adherence, N (%) **					
Adherent	385	(65.5)	407	(64.0)	0.64
Non-adherent	203	(34.5)	227	(35.7)	
					
** Current Smoker, N (%) **					
No	441	(75.0)	531	(83.5)	<0.001
Yes	147	(25.0)	104	(16.4)	
					
** Current Exerciser, N (%) **					
No	259	(44.0)	145	(22.8)	<0.001
Yes	328	(55.8)	486	(76.4)	
					
Participatory Decision-making Score, mean (SD)*	26.0	(5.6)	26.1	(5.0)	0.18
					
** Insurance Type, N (%) **					
Commercial	-	-	248	(39.0)	-
Medicaid	-	-	107	(16.8)	
Medicare	-	-	261	(41.0)	
Uninsured	-	-	20	(3.1)	

Missing values are included in frequency calculations, but not presented in table.

*Participatory decision making score: lowest score = 3; highest score = 30.

**Figure 1:**
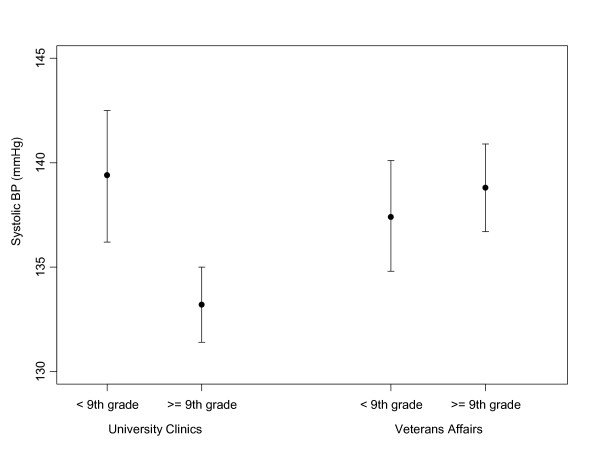
**Adjusted systolic blood pressure by healthcare system and literacy**. Adjusted for variables in model listed on Table 2. Error bars represent 95% confidence intervals. P value from test of interaction between literacy and healthcare system = 0.003.

The results from the SBP multiple linear regression model are presented in Table 2. In the adjusted model, the individual variables that were significantly associated with SBP were patient age, race, and medication adherence. In addition to these main effects, there was a significant interaction between healthcare system and literacy on the outcome of SBP (p = 0.003), suggesting that the relationship between literacy and SBP differed significantly between the two healthcare systems When compared to patients with adequate literacy, the predicted mean SBP for patients with limited literacy was 1.2 mmHg lower in the VAHS (95% CI, -4.8 to 2.3), but 6.1 mmHg higher in the UHS (95% CI, 2.1 to 10.1) (Figure 1). As shown in Table 3, we also observed differences in DBP and BP control according to literacy status in the UHS compared to the VAHS. However, in adjusted analysis the interaction term between literacy and healthcare system was not statistically significant for either of these outcomes (P > 0.05).

**Table 2:** Multiple linear regression model – outcome: systolic blood pressure

**Parameter**	**β Coefficient (95% CI)**	**Standard Error**	**t Value**	**P-value**
**Intercept**	***130.0 (120.0, 140.0)***	***5.1***	***25.5***	***<0.001***

**Main Effects**				
Age, 10 years (continuous)*	1.9 (0.9, 2.9)	0.5	3.8	<0.001
Race (white vs. non-white (ref))	-5.0 (-7.5, -2.6)	1.3	-4.0	<0.001
Currently Married (yes vs. no (ref))	-0.2 (-2.5, 2.2)	1.2	-0.1	0.89
Education (college graduate ref)				
0–9^th ^grade	0.1 (-4.6, 4.8)	2.4	0.05	0.96
10^th^-12^th ^grade	1.0 (-2.1, 4.0)	1.6	0.6	0.54
Some college/vocational tech	1.9 (-1.1, 4.9)	1.5	1.3	0.20
Diabetes (yes vs. no (ref))	-0.7 (-3.1, 1.6)	1.2	-0.6	0.53
Inadequate Income (yes vs. no (ref))	1.3 (-1.6, 4.3)	1.5	0.9	0.37
Medication Adherence (nonadherent vs. adherent (ref))	3.7 (1.3, 6.2)	1.2	3.0	0.003
Current Smoker (yes vs. no (ref))	-2.4 (-5.3, 0.6)	1.5	-1.6	0.11
Current Exerciser (yes vs. no (ref))	1.3 (-1.2, 3.8)	1.3	1.1	0.29
Participatory Decision Making Score (continuous)	-0.1 (-0.3, 0.1)	0.1	-1.0	0.31
**Interaction**				
Literacy by Healthcare System †,	7.4 (2.5, 12.3)	2.5	3.0	0.003
Literacy main effect‡ (REALM < 9^th ^grade vs. ≥ 9^th ^grade (ref))	-1.2 (-4.8, 2.3)	1.8	-	-
Healthcare System main effect‡ (UHS vs. VAHS (ref))	-5.4 (-8.2, -2.6)	1.4	-	-

Total number of patients included in model = 1082.

Positive coefficients represent higher systolic blood pressure; negative coefficients represent lower systolic blood pressure.

* Each ten-year increase in age is associated with a 1.86 mmHg increase in SBP.

† The reference groups for the interaction term are the same as for the main effects.

‡ Main effect of literacy and healthcare system should be interpreted in conjunction with interaction term.

**Table 3:** Mean systolic and diastolic blood pressures and blood pressure control by healthcare system and literacy

	**Healthcare System**			
	***VAHS***		***UHS***	

	***literacy < 9^th^*** ***(n = 226)***	***literacy ≥ 9^th^*** ***(n = 343)***	***literacy < 9^th^*** ***(n = 175)***	***literacy ≥ 9th*** ***(n = 461)***

Mean SBP, (SD)	138.7 (17.8)	138.4 (17.5)	142.5 (24.9)	133.0 (17.6)
Mean DBP, (SD)	75.5 (11.9)	75.5 (11.1)	79.7 (11.8)	77.2 (10.6)
BP in control, n (%)	99 (43.8)	141 (41.1)	76 (43.4)	237 (51.4)

*19 patients were missing a literacy assessment – all from the VAHS

## Discussion

Limited literacy was common in patients with hypertension cared for in both VA and the university affiliated primary care practices. Our study demonstrated that the relationship between literacy and SBP was complex and was conditional on the healthcare system in which patients received their care. More specifically, the relationship between literacy and SBP differed significantly in the two healthcare systems, with much larger differences in SBP according to literacy level for patients in the UHS than the VAHS. The difference in SBP persisted after adjusting for several variables including age, race, medication adherence, and education. In unadjusted analysis, there were similar trends in the relationship between literacy and DBP and BP control according to healthcare system; however the differences were not statistically significant after adjusting for covariates. In spite of this, the observed SBP difference of 6.1 mmHg according to literacy status can contribute to a significant increase in risk for vascular events, especially when considered over many years [30]. These findings suggest that the relationship between literacy and SBP may vary significantly across healthcare systems.

While we did not formally measure any organizational characteristics of either healthcare system, there is reason to believe that the characteristics of the healthcare organization may significantly influence the impact of literacy on health. Changing how healthcare organizations interact with patients with limited literacy has been suggested as a way to improve health disparities related to literacy [31]. Addressing the system of healthcare delivery is a key part of the Chronic Care Model and may be particularly important for patients with limited literacy [32]. Others have shown that literacy may predict response to disease management interventions that change the system of care for these patients [17]. In the growing body of literature examining the relationship between literacy and health outcomes, surprisingly few studies have been conducted in a VA setting and further research in this setting would be helpful to identify whether the relationship between literacy and health differs from other systems.

Although these results have potentially important research and healthcare implications, they should be interpreted with several caveats. The generalizability of our findings may be limited by including only patients sufficiently motivated to participate in a two year randomized controlled trial. In addition, although the combination of two separate datasets provides the advantage of greater statistical power and the ability to compare healthcare systems, these data were collected at different time points from separate randomized controlled trials. To ensure the consistency of our measured variables between studies, we examined the baseline interview from each study carefully and only include variables elicited in the same fashion. Furthermore, the two studies were conducted by the same principal investigator (H.B.B.) and study team, thereby reducing the chance of bias from differential measurement in the two healthcare systems.

An alternative explanation for our findings is that there were systematic differences between the patients in the two healthcare systems that confounded our results. Gender was heavily imbalanced between the two healthcare systems and we were unable to adjust for this variable in the combined model. Also, veterans may adopt different health behaviors as a result of their training in the military that were not captured in our available patient measurements. Although we adjusted for several patient variables that may be associated with systolic blood pressure, we did not include measures of patient knowledge, health beliefs, or health status, which have previously been associated with literacy and may differ between the two patient populations [6,7]. In addition to patient characteristics, we did not explore other variables that may mediate our findings such as type of health insurance coverage or more specific clinic site level differences in how care is delivered.

Finally, although the relationship between literacy and blood pressure significantly differed between these two healthcare systems, further work including a larger number of representative healthcare systems would provide more definitive evidence that literacy's impact on disease outcomes varies across different systems of healthcare delivery. Further work should include more explicit measurement of the financial and organizational characteristics of healthcare delivery. Within a healthcare delivery system, there may be many factors that interact with patient literacy to influence health outcomes. Future studies with more detailed measurement of organizational characteristics are needed to both validate our findings and provide greater information about the factors that may mediate the interaction between literacy and healthcare delivery systems.

## Conclusion

Limited literacy is common in all healthcare delivery systems. This study adds to the existing literature by showing that the relationship between literacy and systolic blood pressure is complex and may differ significantly across different systems of healthcare delivery. This finding is consistent with clinical trials showing that changes in the system of care for chronic disease may influence the relationship between literacy and health outcomes [17,18].

Future studies should include more explicit measurement of the financial and organizational characteristics of healthcare systems to identify the features that may directly interact with literacy to influence health outcomes. Our findings should also be verified in other patient samples as well as with other diseases as the impact of literacy may change as the self-management responsibilities of the patient increase. As we move forward to improve healthcare for our most vulnerable patients, we believe that patients' reading ability and comprehension is a difficult variable to modify. Therefore, a better understanding of the circumstances under which literacy is (or more importantly is not) a contributor to poor health outcomes may be the best way to overcome its impact.

## Competing interests

The authors declare that they have no competing interests.

## Authors' contributions

BJP was responsible for the overall study design and manuscript draft. MKO conducted the statistical analysis and ensured data accuracy. CTT assisted in the analysis approach as well as drafting and editing the manuscript. EZO and HBB were involved in the design and implementation of the two separate clinical trials included in the analysis. Both also provided oversight and direction in the analysis, interpretation, and writing of the manuscript.

## Pre-publication history

The pre-publication history for this paper can be accessed here:


